# A Standardized *Lindera obtusiloba* Extract Improves Endothelial Dysfunction and Attenuates Plaque Development in Hyperlipidemic ApoE-Knockout Mice

**DOI:** 10.3390/plants10112493

**Published:** 2021-11-18

**Authors:** Sang-Hyun Ihm, Sin-Hee Park, Jung-Ok Lee, Ok-Ran Kim, Eun-Hye Park, Kyoung-Rak Kim, Jong-Hoon Kim, Byung-Hee Hwang, Ho-Joong Youn, Min-Ho Oak, Kiyuk Chang

**Affiliations:** 1 College of Medicine, Cardiovascular Research Institute for Intractable Disease, The Catholic University of Korea, Seoul 06591, Korea; limsh@catholic.ac.kr (S.-H.I.); akqmf02@gmail.com (S.-H.P.); okrane@hanmail.net (O.-R.K.); park1119@catholic.ac.kr (E.-H.P.); hbhmac@gmail.com (B.-H.H.); 93015195@cmcnu.or.kr (H.-J.Y.); 2 Division of Cardiology, Department of Internal Medicine, Bucheon St. Mary’s Hospital, The Catholic University of Korea, Seoul 06591, Korea; 3 Division of Cardiology, Department of Internal Medicine, Seoul St. Mary’s Hospital, The Catholic University of Korea, Seoul 06591, Korea; 4 Research and Development Center, Han Wha Pharma, Co., Ltd., Chuncheon 24468, Korea; leejeongok@gmail.com (J.-O.L.); krkim@hwpharm.com (K.-R.K.); 5 Research Center, YangJi Chemicals, Suwon 16229, Korea; jonghoonk@hwpharm.com; 6 College of Pharmacy, Mokpo National University, Muan-gun 58554, Korea

**Keywords:** *Lindera obtusiloba*, apolipoprotein E-deficient mice, atherosclerosis, endothelium

## Abstract

*Lindera obtusiloba* extract (LOE), a traditional herbal medicine used to enhance blood circulation and to reduce inflammation, induced NO-mediated endothelium-dependent relaxation, and reduced the formation of reactive oxygen species (ROS). The study investigated whether LOE improves endothelial dysfunction and reduces plaque inflammation and progression by inhibiting ROS generation in a mouse model of atherosclerosis. Eight-week-old apolipoprotein E-deficient (apoE^−/−^) mice fed with a western diet (WD) were randomized into different groups by administering vehicle (0.5% carboxymethylcellulose (CMC)), LOE (100 mg/kg/day), or losartan (30 mg/kg/day) by gavage until the age of 28 weeks. Fourteen male C57BL/6 mice that were fed normal chow and treated with CMC were used as negative controls. Similar to losartan treatment, LOE treatment induced the concentration-dependent relaxation of aorta rings in WD-fed apoE^−/−^ mice. LOE treatment significantly reduced the vascular ROS formation and expression of NADPH oxidase subunits, including p22phox and p47phox. Compared with WD-fed apoE^−/−^ mice, mice exposed to chronic LOE treatment exhibited reductions in plaque inflammation-related fluorescence signals and atherosclerotic lesions. These effects were greater than those of losartan treatment. In conclusion, LOE treatment improves endothelial dysfunction and reduces plaque inflammation as well as lesion areas by reducing vascular NADPH oxidase-induced ROS generation in a mouse model of atherosclerosis.

## 1. Introduction

Atherosclerosis is a narrowing of the arteries caused by the buildup of plaque. It is characterized by endothelial dysfunction, oxidative stress and chronic low-grade inflammation [1,2,3]. Endothelial dysfunction is the initial event leading to atherogenesis and is associated with increased generation of NADPH oxidase-derived reactive oxygen species (ROS) in vessel walls [4,5]. Increased vascular ROS generation, including hydrogen peroxide and superoxide anions, contributes to the formation of atherosclerotic plaque by inducing oxidative stress, reducing nitric oxide (NO) bioavailability, and promoting proinflammatory responses [3,6,7]. Vascular NADPH oxidase represents an important source of ROS in numerous cardiovascular diseases involving the pathogenesis of atherosclerosis [8,9,10]. Indeed, Nox2 or p47phox protein deficiency reduced the formation of atherosclerotic plaque in high-fat diet-fed apolipoprotein E-deficient (apoE^−/−^) mice [11,12], indicating that NADPH oxidase was crucial for progression of atherosclerotic lesions. Furthermore, inflammation is related to all aspects of atherosclerosis, including the formation of foam cells, the progression and disruption of plaque, and the formation of thrombus [13]. Upregulation of adhesion molecules, cytokines, and chemokines expressed in endothelial cells and their complex interaction promotes leukocyte infiltration into the vascular wall, followed by transendothelial migration, which triggers atherogenesis [14,15].

*Lindera obtusiloba*, which is widely distributed in East Asian countries, has long been used as a traditional herbal medicine to augment circulation in the body, possibly via vasodilation; to suppress inflammation; and to prevent hepatic injury [16]. The extracts were derived from different parts of *L. obtusiloba* and their biologically active compounds such as butenolides, polyphenols, flavonoids, lignans, and neolignans exhibit antioxidant effects. The cytotoxic, anti-allergic, neuroprotective, antithrombotic, and anti-inflammatory effects of these bioactive compounds have been reported [17,18,19,20]. Thus, *L. obtusiloba* possesses an abundance of bioactive compounds, especially antioxidants, which have been studied in several diseases associated with oxidative stress. In addition, the *L. obtusiloba* extract (LOE) inhibits adipogenesis via persistent Wnt signaling and diminishes the tumor necrosis factor α- and lipopolysaccharide-induced IL-6 secretion by preadipocytes, suggesting its therapeutic potential in metabolic syndrome and obesity [21]. Our previous study demonstrated that LOE induces NO-mediated endothelium-dependent relaxation, reduces ROS generation in isolated aortic rings, and prevents hypertension and endothelial dysfunction induced by angiotensin II in rats [22]. In addition, we reported that LOE improves vascular oxidative stress and endothelial dysfunction most likely via normalization of the angiotensin system in diabetic mice [23]. Overall, these findings suggest that LOE has the potential to inhibit ROS generation, to improve endothelial dysfunction in vessel walls, and to reduce inflammatory cytokine production.

Therefore, the aim of the present study was to assess whether LOE improves endothelial dysfunction and prevents the development of atherosclerosis by reducing vascular ROS generation in an experimental model of atherosclerosis, the apoE^−/−^ mice. In particular, the effect of the LOE intake was determined on (1) the endothelium-dependent vascular relaxation of mice aortic rings, (2) the vascular generation of ROS and NADPH oxidase subunits in aortic sections, and (3) the plaque inflammation of aortas and atherosclerotic plaque burden in the aortic sinus.

## 2. Results

### 2.1. LOE Improves Endothelial Dysfunction in WD-Fed apoE^−/−^ Mice

**Figure 1:**
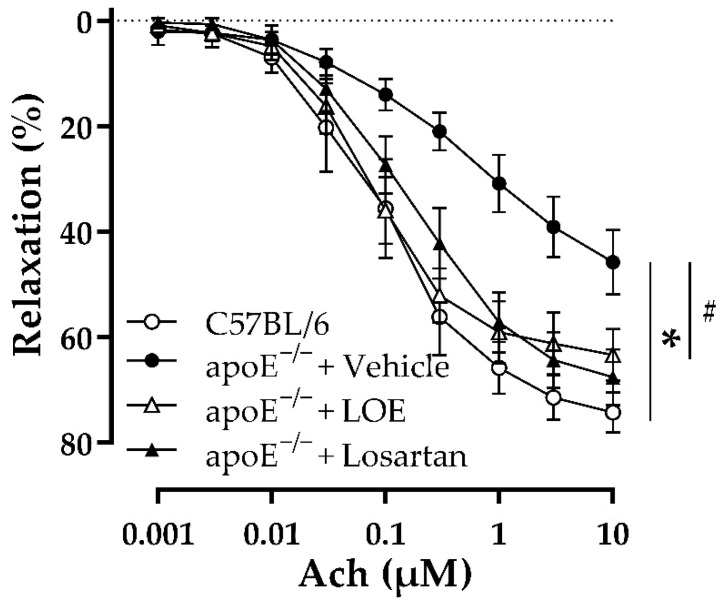
LOE and losartan treatments improve endothelium-dependent relaxation in response to Ach in the aortas of apoE^−/−^ mice. Aortic rings (3–4 mm in length) with endothelium derived from the indicated groups of mice were suspended in organ baths containing oxygenated Krebs solution and precontracted with phenylephrine (100 nM) before the construction of concentration–relaxation curves to Ach (1 nM–10 µM). The results are shown as mean ± SEM (*n* = 5–7). * *p* < 0.05 indicates a significant difference between the apoE^−/−^ vehicle group versus the C57BL/6 group and ^#^ *p* < 0.05 the apoE^−/−^ LOE group or apoE^−/−^ losartan group versus the apoE^−/−^ vehicle group.

The effect of LOE on endothelial dysfunction was investigated by monitoring endothelium-dependent vascular relaxation. Compared with aortic rings isolated from chow diet-fed C57BL/6 mice, those obtained from WD-fed apoE^−/−^ mice showed decreased relaxation to acetylcholine (Ach), suggesting impaired endothelium-dependent vascular relaxation (Figure 1). Both LOE and losartan treatment significantly improved the endothelium-dependent vascular relaxation of aorta rings derived from WD-fed apoE^−/−^ mice (Figure 1).

### 2.2. LOE Decreases Excessive Vascular ROS Formation by Inhibiting NADPH Oxidase Subunits in WD-Fed apoE^−/−^ Mice

**Figure 2:**
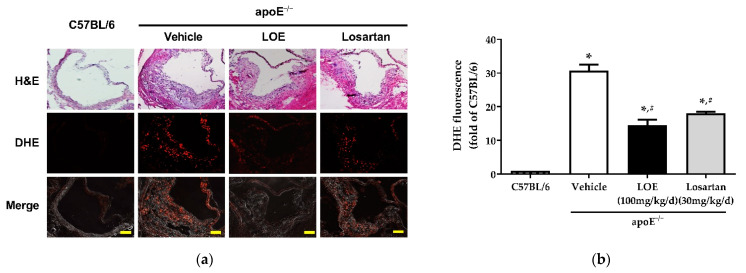
LOE and losartan treatments prevent vascular generation of reactive oxygen species in aortic sections derived from apoE^−/−^ mice. The aortic sections were exposed to dihydroethidine (DHE, 2.5 µM), a redox-sensitive fluorescent dye, for 30 min. Subsequently, ethidium fluorescence was evaluated via confocal microscopy. (**a**) Representative images show H&E staining (**top**), DHE staining in red (**middle**), and merged images (**bottom**). (**b**) Corresponding cumulative data. The scale bar represents 100 µm. The results are shown as mean ± SEM (*n* = 4–6). * *p* < 0.05 versus the C57BL/6 group and ^#^ *p* < 0.05 versus the apoE^−/−^ vehicle group.

To determine the in vivo vascular pro-oxidant effects of LOE therapy, the vascular ROS in the aortic walls of apoE^−/−^ mice and C57BL/6 mice were evaluated using the redox-sensitive fluorescent probe dihydroethidine (DHE). Almost no fluorescence signal was observed in the aortic walls of chow diet-fed C57BL/6 mice (Figure 2). In contrast, there was a significant increase in the DHE fluorescence signal in aortic plaques of WD-fed apoE^−/−^ mice compared with chow diet-fed C57BL/6 mice (Figure 2). Similar to losartan treatment (18.1 ± 0.5 vs. 30.8 ± 1.8, 41.3 ± 1% reduction), LOE administration markedly reduced DHE fluorescence in WD-fed apoE^−/−^ mouse aortas (14.6 ± 1.6 vs. 30.8 ± 1.8 and 52.6 ± 5% reduction) (Figure 2).

**Figure 3:**
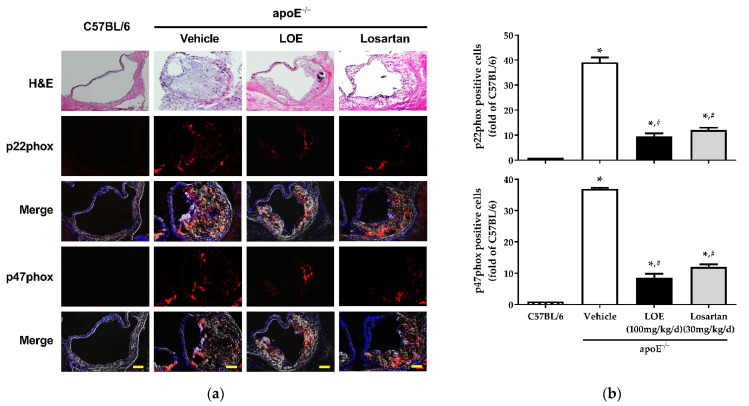
LOE and losartan treatments inhibit NADPH oxidase subunits p22phox and p47phox in aortic sections obtained from apoE^−/−^ mice. The expression of the NADPH oxidase subunits p22phox and p47phox was determined by confocal microscopy using a purified polyclonal antibody and a fluorescence-tagged secondary antibody. Nuclei were stained with DAPI (blue). (**a**) Representative images show H&E staining (**top**), p22phox and p47phox staining in red (**middle**), and merged fluorescence staining and DAPI (**bottom**). (**b**) Corresponding cumulative data. The scale bar represents 100 µm. The results are shown as mean ± SEM (*n* = 4–6). * *p* < 0.05 versus the C57BL/6 group and ^#^ *p* < 0.05 versus the apoE^−/−^ vehicle group.

In addition, since ROS generation in apoE^−/−^ mice has been correlated with the upregulation of NADPH oxidase activity, the activity of NADPH oxidase was assessed based on the expression of its subunits, including p22phox and p47phox [11,24]. The fluorescence signals of p22phox and p47phox were markedly increased in WD-fed apoE^−/−^ mice; however, LOE intake significantly reduced the levels of both NADPH oxidase subunits (p22phox: 9.5 ± 1.1 vs. 39.1 ± 1.9; p47phox: 8.6 ± 1.2 vs. 37 ± 0.3) (Figure 3).

### 2.3. LOE Suppresses Inflammation in Murine Aortic Atherosclerosis

**Figure 4:**
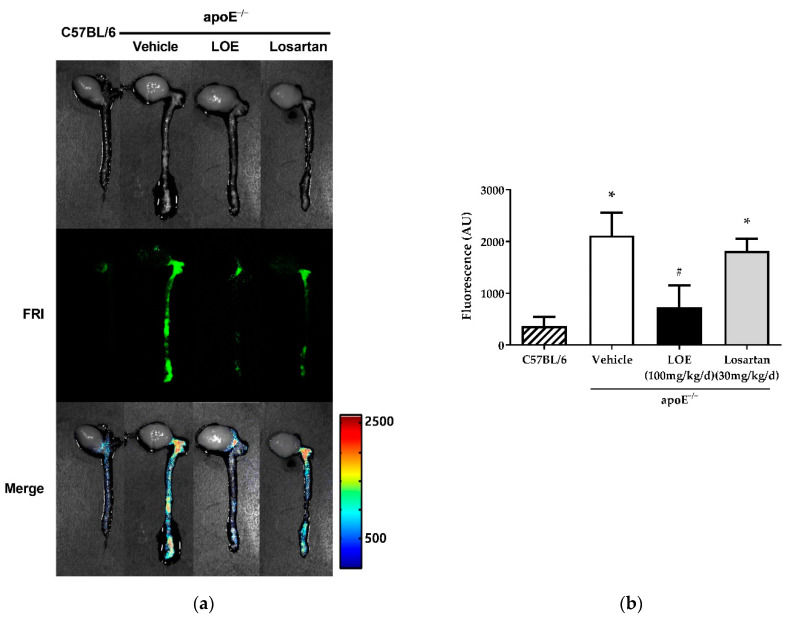
Effect of LOE and losartan treatments on plaque inflammation in the aortas of apoE^−/−^ mice. LOE treatment significantly reduced the degree of plaque inflammation in western diet-fed apoE^−/−^ mice, whereas losartan treatment did not. In vivo imaging of atherosclerotic plaque inflammation was assessed by fluorescence reflectance imaging (FRI) using AP-HGC-Cy5.5 nanoparticles. The extent of plaque inflammation was measured with an IVIS-200 FRI system. (**a**) Middle: FRI; bottom: merged images. (**b**) Corresponding cumulative data. The results are expressed as mean ± SEM (*n* = 4–6). * *p* < 0.05 versus the C57BL/6 group and ^#^ *p* < 0.05 versus the apoE^−/−^ vehicle group.

WD-fed apoE^−/−^ mice exhibited abundant atherosclerotic plaques, especially in the aortic root, arch, and abdominal aorta along the renal artery branches, while atherosclerotic plaques were rarely observed in the aortas of C57BL/6 mice. The plaque inflammation-modulating effects of LOE were assessed via an ex vivo fluorescence reflectance imaging (FRI) study performed to measure inflammation in the aortic atherosclerotic plaques. After 20 weeks of treatment, the aortas of WD-fed apoE^−/−^ mice showed markedly increased inflammation compared with those of C57BL/6 mice (Figure 4). LOE treatment significantly reduced the degree of plaque inflammation in WD-fed apoE^−/−^ mice (594.7 ± 429.8 vs. 1971.0 ± 99.9 AU), whereas losartan treatment did not (Figure 4).

### 2.4. LOE Reduces Atherosclerotic Plaque Burden in apoE^−/−^ Mice in WD-Fed apoE^−/−^ Mice

**Figure 5:**
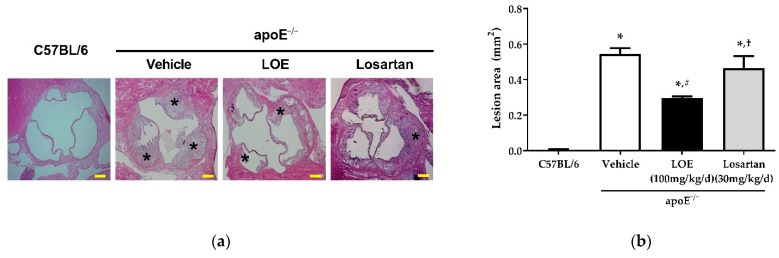
Effect of LOE and losartan treatments on the atherosclerotic plaque areas of aortic sections obtained from apoE^−/−^ mice. LOE treatment significantly reduced the extent of aortic plaque, whereas losartan failed to reduce the area of atherosclerotic lesion in western diet-fed apoE^−/−^ mice. The atherosclerotic plaque area (mm^2^) was measured as the area between the internal elastic lamina and the lumen and quantified by ImageJ after H&E staining. (**a**) H&E staining. Asterisk (*) indicates atherosclerotic plaque. (**b**) Corresponding cumulative data. The scale bar represents 200 µm. The results are expressed as mean ± SEM (*n* = 4–6). * *p* < 0.05 versus the C57BL/6 group, ^#^ *p* < 0.05 versus the apoE^−/−^ vehicle group and ^†^*p* < 0.05 versus the apoE^−/−^ LOE group.

Hematoxylin and eosin (H&E) staining showed atherosclerotic plaques predominantly in the aortic sinus. The plaque area in the aortas of WD-fed apoE^−/−^ mice was increased compared with that of aortas derived from C57BL/6 mice (Figure 5). While losartan failed to reduce the atherosclerotic lesion area in WD-fed apoE^−/−^ mice (0.47 ± 0.07 vs. 0.55 ± 0.03 mm^2^), LOE treatment significantly reduced the aortic plaque area (0.33 ± 0.03 vs. 0.55 ± 0.03 mm^2^) (Figure 5).

## 3. Discussion

The remnants of apolipoprotein B-containing lipoprotein within the arterial walls [25] and subsequent oxidative modification trigger an inflammatory response and endothelial dysfunction [26,27], leading to the formation of atherosclerotic plaques. Current therapeutics against atherosclerotic cardiovascular diseases cannot completely prevent vascular ROS-induced atherogenesis. The major findings of the present study indicate that LOE decreases vascular oxidative stress by suppressing the expression of NADPH oxidase, resulting in improved endothelial dysfunction and in the prevention of atherosclerotic inflammation and lesion progression in a mouse model of atherosclerosis.

A damaged endothelium disturbs the balance between vasoconstrictors and vasodilators and leads to multiple events that promote or exacerbate atherosclerosis [28]. Several studies have suggested that a number of polyphenol-rich natural products are capable of improving endothelium-dependent relaxation by enhancing the endothelial production of vasoprotective factors including NO and EDH and ultimately prevent endothelial dysfunction in cardiovascular diseases including hypertension [29,30,31]. Our previous studies revealed that LOE was a potential vasorelaxant acting via NO, and that LOE-induced relaxation was attenuated by PI3-kinase/Akt pathway inhibitors in isolated aortic rings. LOE induced a time-dependent phosphorylation of Akt at Ser473 and eNOS at Ser1177 in endothelial cells [22]. The findings suggest that LOE was an activator of the PI3-kinase/Akt-dependent eNOS phosphorylation at Ser 1177. In addition, LOE treatment attenuated endothelial dysfunction and hypertension induced by angiotensin II in rats [22]. Long-term administration of LOE to diabetic mice restored the abolished endothelium-dependent relaxation to Ach in aortic rings and ameliorated hyperglycemia partially [23]. The current findings based on aortic rings isolated from apoE^−/−^ mice, an experimental model of atherosclerosis, clearly indicate that chronic LOE treatment improved endothelium-dependent vascular relaxation to Ach in WD-fed apoE^−/−^ mice to a degree comparable to losartan. Losartan is a well-known angiotensin II receptor antagonist with anti-hypertensive activity and is used to treat hypertension and heart failure. Previous studies have shown that losartan exhibits anti-atherosclerotic effects in apoE^−/−^ mice by reducing lipid accumulation and macrophage infiltration as well as by inhibiting LDL lipid peroxidation [32,33].

Endothelial dysfunction is triggered by high levels of NADPH oxidase-derived superoxide anions, which react with NO, thereby decreasing its bioavailability in the arterial wall. NADPH oxidase, which was detected in neutrophils, is found in vascular endothelial cells and smooth muscle cells, and essential source of superoxide formation in several animal models of vascular disease [34]. Furthermore, p22phox, one of the NADPH oxidase components, has been identified in atherosclerotic coronary arteries of humans [35]. In this study, we demonstrated that LOE inhibited the increased levels of vascular oxidative stress in apoE^−/−^ mice as indicated by the pronounced DHE staining in all of the aortic plaques and the arterial wall as well as the excessive vascular expression of NADPH oxidase subunits, including p22phox and p47phox. The findings indicate that the potential effect of LOE intake on endothelium-dependent vascular relaxation is most likely associated with its ability to reduce vascular oxidative stress, partially via decreasing the NADPH oxidase expression in apoE^−/−^ mice. Indeed, previous studies suggested that tea and grape-derived polyphenols downregulate the expression of NADPH oxidase subunits such as p22phox and nox1, and tea polyphenols induce upregulation of catalase expression in vascular cells [29,36]. Since endothelial dysfunction is identified ahead of structural alterations in the arterial wall, it most likely acts as an early signaling event in the pathological initiation and progression of atherosclerosis [28].

Inflammation, which is mediated by various factors such as cytokines, adhesion molecules, and NO, is considered a vital component of atherogenesis [1,3]. Several studies have shown that dietary polyphenols, especially theaflavin and quercetin, ameliorate atherosclerosis by improving inflammation and bioavailability of NO in apoE^−/−^ mice [37,38]. We employed molecular FRI as a sensing platform to measure plaque inflammation after injecting AP-HGC-Cy5.5 nanoparticles as molecular imaging agents. These nanoparticles selectively distinguish atherosclerotic plaques by binding to the IL-4 receptor on macrophages, endothelial cells, and smooth muscle cells. Therefore, this molecular imaging tool facilitates the visualization of early atherosclerotic plaque lesions [39]. We detected that increased plaque inflammation in WD-fed apoE^−/−^ mice and LOE treatment for 20 weeks resulted in marked regression of plaque inflammation, even more than losartan treatment. A reduction in plaque inflammation in LOE-treated apoE^−/−^ mice decreased the aortic plaque area, suggesting anti-atherogenic and anti-inflammatory effects of LOE in apoE^−/−^ mice.

## 4. Materials and Methods

### 4.1. Plant Extraction and Standardization

Plant extraction and standardization were performed as described previously [22]. Briefly, the *L. obtusiloba* stems were collected in the vicinity of Hongcheon, Korea, and the voucher specimen (no. YJP-14) was stored at the Herbarium of KIST Gangneung Institute (Gangneung, Korea). *L. obtusiloba* was identified by Dr. Sang Hoon Jung, KIST Gangneung Institute, Gangneung, Korea. Short and dried branches were segmented into small slices and pulverized with a commercially available food mixer. Using 50% ethanol, 15.8 kg of *L. obtusiloba* powder was extracted four times for 4 h at 70 °C. The ethanolic solution was vaporized to dryness under vacuum to obtain 1.7 kg of the total extract (yield 7.0%). The polyphenol content in the LOE was evaluated via the Folin–Ciocalteau assay and was approximately 23.7% described as (−)-epicatechin equivalents. Previous study has reported that the major compounds in LOE were hyperin, isoquercitrin, guaijaverin, avicularin, and quercitrin [22,23]. Among them, hyperin and isoquercitrin were determined as standard substances for standardization of LOE. An HPLC analysis of LOE yielded a linear calibration curve for the standard compounds, which include hyperin and isoquercitrin. The main compounds including hyperin and isoquercitrin were identified in *L. obtusiloba* stems and designated as standard compounds by the Korea Food and Drug Administration (KFDA). LOE (10 μL) in 50% aqueous methanol (10 mg/mL), hyperin (16 μg/mL), and isoquercitrin (7 μg/mL) was loaded onto an Agilent 1200 series HPLC system. Separation was performed using a C18 reverse-phase column (4.6 × 250 mm, 10 μm, Shiseido, Tokyo, Japan) at 35 °C and eluted at a flow rate of 1.0 mL/min using a mobile phase of 17% aqueous acetonitrile acidified with 0.1% trifluoroacetic acid. Chromatographic profiles were recorded at 254 nm. The correlation coefficient was 0.998 for the respective standard curve. Hyperin and isoquercitrin in the LOE were identified and quantified at 15.33 min and 16.48 min, respectively, as shown in Supplementary Figure S1. The results revealed that 100 mg of LOE contains 0.138 mg of hyperin and 0.055 mg of isoquercitrin. These concentrations are within the appropriate ranges approved by the KFDA. All other quality control test results including heavy metals and pesticide residues were within acceptable limits.

### 4.2. Animals

This study complied with the Guide for the Care and Use of Laboratory Animals published by the US National Institutes of Health (NIH Publication No. 85-23, revised 1996) and was approved by the IACUC of Gyeonggi Biocenter (Approval No. 2009-09-09). For this study, 14 male C57BL/6 mice (6 weeks old, 21–24 g) and 42 male apoE^−/−^ mice (6 weeks old, 20–25 g) were purchased from Central Lab (Seoul, Korea) and acclimated with normal chow diet for 2 weeks. At the age of 8 weeks, apoE^−/−^ mice were randomized into 3 groups; fed a western diet (WD: Research Diets, Inc. (New Brunswick, NJ, USA) composed of (wt/wt) 20% protein, 50% carbohydrate, 21% fat, and 0.15% cholesterol); and administered the vehicle (0.5% carboxymethylcellulose (CMC); apoE^−/−^ vehicle group, *n* = 14), LOE (100 mg/kg per day; apoE^−/−^ LOE group, *n* = 14), or losartan (30 mg/kg per day; apoE^−/−^ losartan group, *n* = 14) by gavage until the age of 28 weeks. C57BL/6 mice exposed to a normal chow diet and treated with 0.5% CMC by gavage were used as negative controls. The dose of losartan was selected based on previous studies indicating that a maximal functional effect without hemodynamic compromise can be obtained with this dose (data not shown). After 20 weeks of treatment, blood was collected under pentobarbital (50 mg/kg, i.p.) anesthesia, and the aorta and the heart were removed. The blood was centrifuged (3000 rpm, 10 min), and serum was obtained and stored at −70 °C until use.

### 4.3. Vascular Reactivity

Aortic rings (3–4 mm in length) were obtained and mounted in myographs and placed in organ baths containing Krebs bicarbonate solution (in mM: 119 NaCl, 4.7 KCl, 1.18 KH_2_PO_4_, 1.18 MgSO_4_, 1.25 CaCl_2_, 25 NaHCO_3_, and 11 D-glucose, pH 7.4), which was oxygenated (95% O_2_; 5% CO_2_) and warmed to 37 °C to measure isometric tension. Following equilibration for 60 min under a resting tension of 1.0 g, the maximal contraction was measured by monitoring vasoconstriction evoked using a potassium-rich Krebs solution (80 mM) for 10 min. Subsequently, the rings were washed for 60 min and contracted with phenylephrine (100 nM). After a washout and a 30-min equilibration period, the rings were contracted again with increasing concentrations of phenylephrine to approximately 80% of the maximal contraction. The relaxation induced by cumulative treatment with Ach (1 nM–10 µM) on a half-logarithmic scale was measured to yield a concentration-relaxation curve to Ach. Concentration response is expressed as a percentage of contraction by phenylephrine.

### 4.4. Determination of Vascular ROS Formation

The in situ ROS synthesis was measured using the oxidative fluorescent dye DHE (Sigma–Aldrich, Milwaukee, WI, USA) as previously described [4]. Thoracic aortas from all groups were embedded into an optimal cutting temperature (OCT) compound (O.C.T. Tissue-Tek, Sakura Finetek, Torrance, CA, USA) and then frozen in liquid nitrogen for cryostat sectioning. The frozen aortas were sliced into 5 μm thick sections, followed by incubation with DHE (2.5 μM) in a humidified light-protected chamber for 30 min at 37 °C. Images were examined using a confocal microscope (LSM 510 META, Carl Zeiss, Inc., Overkochen, Germany) with a 20× epifluorescence objective. Mean intensities are expressed as arbitrary densitometric units.

### 4.5. Immunohistochemical Analysis of NADPH Oxidase Subunit Expression

Thoracic aorta sections (5 μm) were sliced from paraffin blocks fixed with 4% paraformaldehyde. Antigen retrieval was performed by heating the sections in a 60 °C oven with a 10 mM citrate buffer overnight. After cooling, the sections were incubated with 3% hydrogen peroxide for 10 min to inhibit endogenous peroxidases. A rabbit polyclonal anti-p22phox antibody (Santa Cruz Biotechnology, Santa Cruz, CA, USA) was used at a 1:50 dilution for p22phox immunostaining. An Alexa Fluor 488-conjugated secondary antibody (Invitrogen Corp., Carlsbad, CA, USA) was used at a 1:2000 dilution. For p47phox immunostaining, the sections were incubated with a rabbit polyclonal anti-p47phox antibody (Santa Cruz Biotechnology, Santa Cruz, CA, USA) diluted at 1:50 in 0.5 M TBST at 4 °C overnight. An Alexa Fluor 555-conjugated secondary antibody (Invitrogen Corp., Carlsbad, CA, USA) was used at a 1:2000 dilution. Nuclear counterstaining was accomplished with DAPI at a 1:10,000 dilution. The sections were stored in the dark until they were analyzed using a confocal microscope (LSM 510 META, Carl Zeiss, Inc., Overkochen, Germany).

### 4.6. Macroscopic Fluorescence Reflectance Imaging of Plaque Inflammation

To investigate the ability of LOE to reduce plaque inflammation in apoE^−/−^ mice, we employed ex vivo FRI to evaluate aortic plaque inflammation. Twenty-four hours before imaging, molecular imaging agents targeted against inflammatory endothelial cells (atherosclerotic plaque-homing peptide (AP)-hydrophobically modified glycol chitosan (HGC)-Cy5.5 nanoparticles; kindly provided by Dr. KM Kim from KIST (Korea)) were intravenously injected via the tail vein (10 mg/kg) [39]. The extent of plaque inflammation in apoE^−/−^ mice compared with control C57BL/6 mice and the plaque inflammation-modulating effects of LOE compared with losartan were determined via the ex vivo FRI of the extracted aortas. Immediately before imaging, the mice were sacrificed via cervical dislocation and perfused with 20 mL saline. The aortas, which were connected to the hearts, were then excised and imaged in the Cy5.5 channel on an IVIS-200 FRI system (Xenogen Corp., Alameda, CA, USA).

### 4.7. Measurement of H&E Staining and the Aortic Atherosclerotic Plaque Area

After sacrifice, the mice were perfused with PBS through the left ventricle. For histopathological analysis, isolated aortic roots and right carotid arteries were embedded in an OCT compound. The frozen sections of the embedded aortic roots and carotid arteries were obtained. Adjacent sections were stained with H&E for general morphological analysis. The aortic lesion area (mm^2^) was quantified using ImageJ software (NIH). The images of aortic plaque were calibrated using a hemocytometer, with 1 mm considered equal to 1600 pixels. The aortic plaque area was estimated as the area between the internal elastic lamina and the lumen on H&E sections.

### 4.8. Statistical Analysis

Statistical analysis was performed using SPSS software (version 11, SPSS, Inc., Chicago, IL, USA). All values are reported as the mean ± SEM. Differences in the measured values among multiple groups were analyzed via analysis of variance, followed by Bonferroni’s multiple comparison. For all statistical analyses, a *p*-value less than 0.05 was considered statistically significant.

## 5. Conclusions

Treating WD-fed apoE^−/−^ mice with LOE improves endothelial dysfunction by reducing NADPH oxidase expression and the formation of ROS. Consequently, LOE treatment is associated with the prevention of atherosclerotic inflammation and plaque development in apoE^−/−^ mice. Altogether, the present findings indicate that LOE might be an attractive herbal candidate for the development of atherosclerosis associated with endothelial dysfunction and vascular oxidative stress.

## Data Availability

All the data generated and analyzed during this study are included in this article.

## Supplementary Materials

The following are available online at https://www.mdpi.com/article/10.3390/plants10112493/s1, Figure S1: Representative HPLC chromatograms of hyperin and isoquercitrin (a), and LOE (b) under conditions described in the Materials and Methods section. Peaks for hyperin and isoquercitrin are indicated.
