# Supplementary material for: A Standardized Lindera obtusiloba Extract Improves Endothelial Dysfunction and Attenuates Plaque Development in Hyperlipidemic ApoE-Knockout Mice
Source: Plants (Basel). 2021 Nov 18;10(11):2493. doi: 10.3390/plants10112493 (PMC8618780; doi:10.3390/plants10112493)
Supplement: Supplementary file 1 [file plants-10-02493-s001.zip › plants-1450792-supplementary.pdf]

Supplementary Figure S1

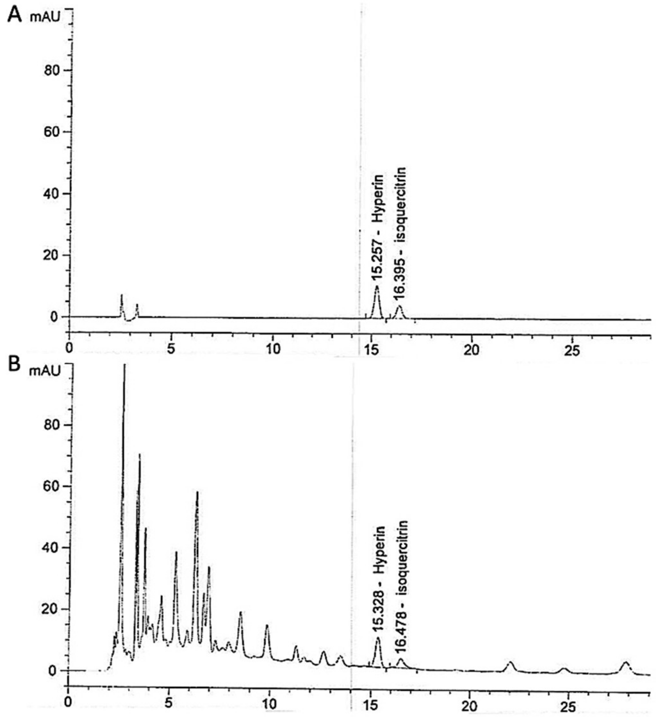

Supplementary Figure S1. Representative HPLC chromatograms of hyperin and isoquercitrin (**A**) and LOE (**B**) under conditions described in the Materials and Methods section. Peaks for hyperin and isoquercitrin are indicated.
